# The relationship between foot posture index, ankle equinus, body mass index and intermetatarsal neuroma

**DOI:** 10.1186/s13047-016-0179-9

**Published:** 2016-12-01

**Authors:** Reza Naraghi, Alexandra Bremner, Linda Slack-Smith, Alan Bryant

**Affiliations:** 1 School of Surgery, Podiatric Medicine Unit M422, The University of Western Australia, 35 Stirling Highway, Crawley, WA 6009 Australia; 2 School of Population Health M431, The University of Western Australia, 35 Stirling Highway, Crawley, WA 6009 Australia; 3 School of Dentistry M512, The University of Western Australia, 35 Stirling Highway, Crawley, WA 6009 Australia

**Keywords:** Neuroma, Equinus deformity, Body mass index

## Abstract

**Background:**

The main purpose of this study was to investigate the presence of an association between intermetatarsal neuroma and foot type, as measured by the Foot Posture Index. The study also examined whether there was a relationship between foot type and the interspace affected with intermetatarsal neuroma, and whether ankle equinus or body mass index had an effect.

**Methods:**

In total, 100 participants were recruited from The University of Western Australia’s Podiatry Clinic, 68 of whom were diagnosed with inter-metatarsal neuroma from 2009 to 2015. There were 32 control participants recruited from 2014 to 2015. The age of subjects was recorded, as were weight and height, which were used to calculate body mass index. The foot posture index and ankle dorsiflexion were measured using standard technique. Independent t-tests and Kruskal-Wallis tests were used to compare differences in foot posture index, body mass index and ankle dorsiflexion between the inter-metatarsal neuroma and control groups. Multivariable logistic regression was also used to model relationships for outcome.

**Results:**

The 68 intermetatarsal neuroma subjects had a mean age of 52 years (range 20 to 74 years) and comprised of 56 females and 12 males. The 32 control subjects had a mean age of 49 years (range 24 to 67 years) with 26 females and six males. There were no significant differences between the control and the intermetatarsal neuroma groups with respect to the mean foot posture index scores of the left and right foot (*p =* 0.21 and 0.87, respectively). Additionally no significant differences were detected between the affected intermetatarsal neuroma interspace and foot posture index (*p* = 0.27 and 0.47, respectively). There was no significant difference in mean body mass index between the intermetatarsal neuroma (26.9 ± 5.7) and control groups (26.5 ± 4.1) (*p* = 0.72). There was, however, a significant difference in mean ankle dorsiflexion between the intermetatarsal neuroma and control groups (*p* < 0.001 for both feet). Logistic regression models, adjusted for age, sex, foot posture index and body mass index estimated that the odds of having an intermetatarsal neuroma in the right foot increased by 61% (OR 1.61; 95% CI 1.32–1.96) with each one degree reduction of ankle dorsiflexion, and in the left foot by 43% (OR 1.43; 95% CI 1.22–1.69).

**Conclusion:**

No relationships were found between foot posture index and body mass index with intermetatarsal neuroma, or between foot posture index and the interspaces affected. However, a strong association was demonstrated between the presence of intermetatarsal neuroma and a restriction of ankle dorsiflexion.

## Background

Intermetatarsal neuroma (IMN), also known as Morton’s metatarsalgia is the symptomatic thickening of the plantar intermetatarsal nerve at the level of bifurcation into the digital branches. It is more common in women, and the highest hospital admission rates for surgical removal are for 55–59 year-old males and 50–55 year-old females [1]. People with IMN complain of a sharp burning pain in the interspaces and tingling sensations that may radiate to the toes. This condition commonly affects the third interspace, however, neuromas in the second interspace are also common, while the first and fourth interspaces are rarely involved [2–6].

There are numerous aetiologies speculated in the literature for IMN, such as; pronation [4, 7, 8], metatarsus proximus [15, 16], trauma [9], ankle equinus [10–14], bursitis [9, 15, 16], entrapment by the deep transverse metatarsal ligament [9, 17], and anatomical variations such as presence of the communicating branch of the lateral plantar nerve [7, 18, 19]. Jarde reported that flatfoot was associated with the development of IMN in 44% of a 43 patient series [20]. Hagedorn et al., in a 2013 study of 3429 participants, reported associations between foot posture and common foot problems such as hallux abducto valgus, hammertoe, overlapping toe, hallux rigidus and IMN [21]. Their results showed no association between IMN (*N* = 439) and any foot posture and function. However, their study did not compare the foot posture of subjects with foot disorders to that of control subjects.

Excessive pronation can lead to hypermobility of the metatarsal heads, and it has been postulated that movement between the fixed medial column and the more mobile lateral column of the foot can place excessive pressure on the third interspace nerve [4, 7, 15, 20, 22, 23]. This, along with the traction caused by the flexor digitorum brevis has been implicated as a possible cause of the formation of neuroma in the third interspace [4]. It has been suggested that neuroma in the second interspace is more common in the neutral to cavus foot due to the close approximation of the second and third metatarsal heads [24]. This tight space predisposes the nerve to compression by the bursa above the nerve and lumbricalis muscle/tendon arising from the medial aspect of the flexor digitorum longus that runs parallel to the nerve [25, 26]. Furthermore, the plantar declination of the metatarsals in a cavus foot type can increase pressure over the corresponding nerve [27]. Pazzaglia et al. reported that 75% of his IMN patients (*n* = 12) in an immunohistochemical study had a cavus foot type with forefoot deformity [6]. There were no case-control studies in the literature that investigated the association of IMN with foot posture. Additionally no proposed mechanism that related foot posture to the occurrence of IMN in the second and third interspaces was identified. This lends us to hypothesize that the occurrence of neuroma in the third interspace would be associated with a pronated foot posture and the occurrence of neuroma in the second interspace would be present in a more neutral to supinated foot type.

To measure the association of foot posture and IMN, investigators can use a simple and efficient tool such as The Foot Posture Index ™ (FPI). FPI is regularly used by clinicians to assess foot type prior to implementing orthotic therapy [28]. The FPI measurement tool has also been validated using Rasch analysis, “which showed that it had good psychometric properties, good individual item fit, and good overall fit of the six criteria to the obtained model” [29]. In addition, Cornwall et al. showed that the FPI had high intra-rater reliability with intraclass correlation coefficient (ICC) levels of greater than 0.9, however, the inter-rater reliability with ICC values were moderate between 0.525 and 0.655 [30]. Although there is no available literature that has investigated the FPI and IMN, this tool is frequently reported for association of foot type with many other lower extremity conditions [31–33].

Reduced ankle joint dorsiflexion also known as ankle equinus is surmised to cause IMN [10, 13, 14, 34, 35]. A lack of adequate ankle dorsiflexion can result in compensation during gait such as; an early heel lift and an increase in forefoot pressures [11] and thus causing pain in the forefoot [14]. Measurement of ankle joint dorsiflexion is used frequently by clinicians in their day to day practice. There is limited high quality evidence to support the relationship between ankle joint range of motion and IMN, only one case study by Barrett and Jarvis reported an improvement to forefoot nerve symptoms after a gastrocnemius release [35].

This study investigates the association between foot type as measured by the FPI, ankle equinus and body mass index (BMI) and the presence of IMN. This study examines the relationship between foot type and the affected interspace with neuroma.

## Methods

As a case-control study, subjects were recruited from patients attending The University of Western Australia (UWA) Podiatry Clinic. The inclusion criteria for IMN subjects included a minimum of 6-month history of pain in an affected interspace and a clinically demonstrated positive painful Mulder’s click and a positive ultrasound confirmatory diagnosis of neuroma in the affected interspace. The inclusion criterion for control subjects was no history of IMN or neuroma-like pain in the forefoot. The exclusion criteria for both neuroma and control groups were a previous history of surgery to the lower extremity, any proximal nerve entrapment at the level of the ankle, knee, hip or back, any history of significant trauma to the forefoot area, any difficulty in walking and standing, diabetes or a history of systemic arthritis, bony ankle equinus and any other cause of pain in the forefoot such capsulitis/tenosynovitis or plantar plate pathology.

### Recruitment

Approval was obtained from the University of Western Australia Human Research Ethics Committee for this study, which recruited 100 participants from the UWA Podiatry Clinic, 68 of whom were diagnosed with IMN from 2009 to 2015. There were 32 control participants recruited from 2014 to 2015. All participants provided a medical history and underwent a physical examination by the corresponding author.

### Measurements

Subjects’ ages, weights and heights were recorded, and their BMIs calculated. The FPI was measured according to the FPI User Guide Manual [36] by the corresponding author, who has more than 10 years of experience in clinical practice. Measurements were taken twice and the average value was recorded. Ankle dorsiflexion was measured for each subject with a goniometer using the technique described by Root et al. [37]. The subtalar joint was placed in neutral with the patient in a prone position and the ankle dorsiflexed passively while maintaining subtalar joint neutral position. The subject was then asked to actively dorsiflex the foot while the examiner maintained the subtalar joint in a neutral position. The angle formed between the lateral rear foot and the lateral bisection of the distal 1/3 of the fibula was measured. Two measurements were taken and the average recorded. The intra-rater reliability of the ankle dorsiflexion measurements was tested by using the measurements of eight subjects performed three times to calculate the ICC using a two-way mixed effects model in IBM SPSS Statistics v22 (IBM Corp, Armonk, NY, USA). The ICC of 0.95 (95% CI 0.83–0.99) indicated that intra-rater reliability was good.

### Statistical analysis

IBM SPSS Statistics v22 was used for analyses. The significance level was set at 0.05. Results are expressed as mean ± SD. Medians and ranges are also presented for non-normally distributed measures. Independent sample *t*-tests were used to compare the mean age, BMI, FPI and ankle dorsiflexion between IMN and control groups. In addition, Kruskal-Wallis tests were performed to test for differences in foot type and ankle dorsiflexion between the interspaces affected. Chi-square tests were used to determine whether there was any association between the interspace(s) and the foot (feet) affected in IMN subjects, and whether the proportions of males and females with ankle dorsiflexion of less than 10° differed. Associations were also investigated using multivariable logistic regression. Odds ratios (ORs) and 95% confidence intervals (CIs) are reported.

## Results

The 68 IMN cases had a mean age of 52 ± 14 years (range 20 to 74 years) and comprised 56 females and 12 males. The control group of 32 subjects had a mean age of 49 ± 10 years (range 24 to 67 years), with 26 females and six males. There were no significant differences in age between the IMN and control groups (*p* = 0.28), or BMI between the IMN (26.9 ± 5.7) and control (26.5 ± 4.1) groups (*p* = 0.72). Approximately equal percentages of men and women had IMN: 66.7% of men and 68.3% of women (*p* = 0.89).

**Fig. 1:**
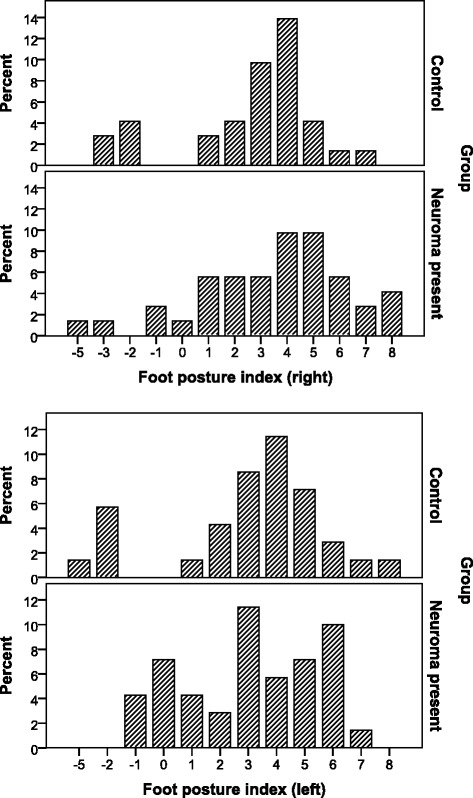
Graph of foot posture index for intermetatarsal neuroma and control groups – *left* and *right* feet

**Fig. 2:**
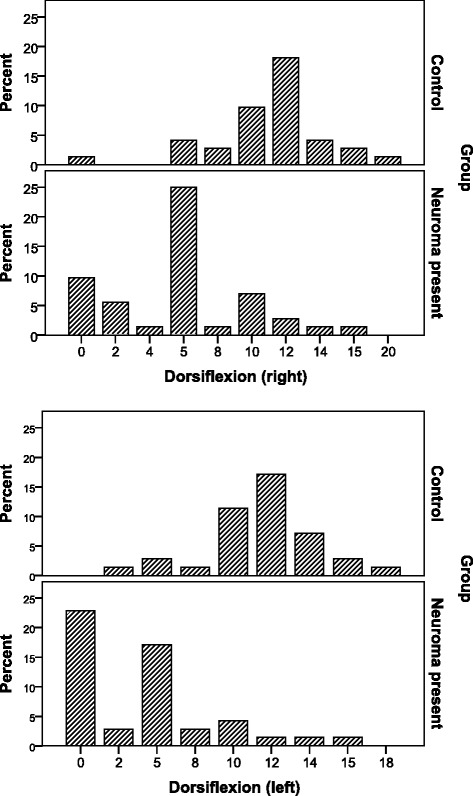
Graph of ankle dorsiflexion in degrees for intermetatasral neuroma and control groups - *left* and *right* feet

Figure 1 shows the FPI scores for the IMN and control groups. The mean FPI scores were 3.5 ± 2.9 (range -5 to 8) for the right-foot IMN and 2.9 ± 2.8 (range -1 to 7) for the left-foot IMN (Table 1). The control group mean FPI score for the right foot was 2.7 ± 2.5 (range -3 to 7) and for the left foot, 3.0 ± 2.9 (range -5 to 8). There were no significant differences in the mean FPI scores for the right and left feet between cases and controls (*p* = 0.21 and 0.87, respectively). There were, however, significant differences in mean ankle dorsiflexion between the IMN and control groups (*P* < 0.001 for both feet). The ankle dorsiflexion measurements of IMN subjects were lower by 5.91° (95% CI 4.04–7.78) for the right foot, and 7.34° (95% CI 5.55–9.13) for the left foot. Figure 2 shows the ankle dorsiflexion measurements of the IMN and control groups. Male and female ankle dorsiflexion measurements did not differ significantly, nor did the proportions of male and female IMN subjects with ankle dorsiflexion less than 10 degrees. They were 87.5% versus 75.0% on the right (*p* = 0.66), and 75.0% versus 86.7% on the left (*p* = 0.59), for males and females, respectively.

**Table 1:** Comparison of characteristics between intermetatarsal neuroma and control groups by foot affected

		Case (IMN present) *n* = 68		Control (No IMN) *n* = 32		
		Mean ± SD	Range	Mean ± SD	Range	*p*-value
IMN right foot *n* = 40	FPI	3.5 ± 2.9	-5 to 8	2.7 ± 2.5	-3 to 7	0.210
	ADF	5.33 ± 3.99	0 to 15	10.90 ± 3.62	0 to 20	<0.001
IMN left foot *n* = 38	FPI	2.9 ± 2.8	-1 to 7	3.0 ± 2.9	-5 to 8	0.870
	ADF	4.10 ± 3.89	0 to 15	11.30 ± 3.14	2 to 18	<0.001

*IMN* Intermetatarsal neuroma, *FPI* Foot Posture Index, *ADF* Ankle dorsiflexion (degrees)

*P*-values from independent *t*-test comparison of cases with controls. Note there is some overlap in cases as some subjects have IMN in both feet

Of the IMN subjects, 28 were diagnosed with neuroma in the second interspace, 23 in the third interspace and 17 in both 2^nd^ and 3^rd^ interspaces. Only three subjects had neuromas in the second and third interspaces of both feet (Table 2). There was no significant association between the interspace(s) affected and the foot (feet) affected with IMN(s) (*p* = 0.16).

**Table 2:** Description of intermetatarsal neuroma by foot and the interspace(s) affected

Interspace	Right foot	Left foot	Both feet	Total
2/3	14	9	5	28 (41.2%)
3/4	12	8	3	23 (33.8%)
Both	3	11	3	17 (25.0%)
Total	29 (42.6%)	28 (41.2%)	11 (16.2%)	68

Total interspaces affected: right foot (*n* = 40); left foot (*n* = 38)

In order to evaluate the relationship between FPI and interspaces affected the IMN subjects were divided according to their affected interspace and compared with controls. The second interspace FPI means on the right (*n* = 19) and left (*n* = 13) were 3.2 ± 2.6 and 2.7 ± 1.9, respectively. The third interspace FPI means on the right (*n* = 15) and left (*n* = 11) were 3.20 ± 3.5 and 2.9 ± 2.8, respectively (Table 3). FPI did not differ significantly across groups when IMN subjects were divided according to their affected interspace(s) (*p* = 0.27 on the right and *p* = 0.47 on the left) nor did ankle dorsiflexion differ across interspace groups (*p* = 0.80 on the right and *p* = 0.79). Logistic regression models, adjusted for age, sex, FPI and BMI, estimated that the odds of having an intermetatarsal neuroma in the right foot decreased by 38% (OR 0.62; 95% CI 0.51–0.76) with each additional degree of ankle dorsiflexion, and in the left foot by 30% (OR 0.70; 95% CI 0.59–0.82) (Table 4). Alternatively, these odds ratios can be expressed for each degree of reduction in ankle dorsiflexion as 1.61 (95% CI 1.32–1.96) for the right foot and 1.43 (95% CI 1.22–1.69) for the left foot. That is, the odds of having a neuroma increased by 61 and 43% for the right and left feet, respectively, with each degree of reduction in ankle dorsiflexion.

**Table 3:** The relationship between the affected interspaces and foot posture index and ankle dorsiflexion

	Right foot			Left foot		
Interspace	n	FPI mean ± SD median (range)	ADF mean ± SD median (range)	n	FPI mean ± SD median (range)	ADF mean ± SD median (range)
2/3	19	3.2 ± 2.6 4 (-3 to 7)	4.63 ± 2.87 5 (0 to 12)	13	2.7 ± 1.9 3 (0 to 5)	3.62 ± 3.84 2 (0 to 10)
3/4	15	3.2 ± 3.5 3 (-5 to 8)	5.87 ± 4.66 5 (0 to 14)	11	2.9 ± 2.8 3 (-1 to 7)	4.55 ± 4.25 5 (0 to 12)
Both	6	5.2 ± 1.7 5 (3 to 8)	6.17 ± 5.49 5 (0 to 15)	14	3.6 ± 2.5 4 (-1 to 6)	3.86 ± 5.10 2.5 (0 to 15)
*p*-value		0.27	0.80		0.47	0.79

*p*-value from Kruskal-Wallis one way analysis of variance test (non-parametric test)

**Table 4:** Multivariable logistic regression estimates of odds of one or two intermetatarsal neuromas

	Right foot		Left foot	
Variable	OR (95% CI)	*p*-value	OR (95% CI)	*p*-value
Age (years)	1.06 (0.99, 1.14)	0.75	0.99 (0.94, 1.04)	0.75
BMI (kg/m2)	0.88 (0.73, 1.06)	0.20	1.09 (0.96, 1.24)	0.20
Female^a^	2.08 (0.34, 12.8)	0.95	0.96 (0.23, 4.03)	0.95
FPI	1.29 (0.93, 1.81)	0.85	1.02 (0.82, 1.27)	0.85
ADF (degrees)	0.62 (0.51, 0.76)	<0.001	0.70 (0.59, 0.82)	<0.001

*BMI* Body mass index

*FPI* Foot Posture index

*ADF* Ankle dorsiflexion (degrees)

*OR* Odds ratio, *CI* Confidence interval

^a^Compared to male. Other ORs are per unit increase

In summary, there were no significant differences in FPI between IMN interspaces affected or between cases and controls. The IMN subjects had a significant decrease in ankle dorsiflexion measurements compared with control subjects.

## Discussion

This study used the FPI to investigate for an association between foot posture and IMN. The results showed no association between foot posture and IMN formation. Our mean FPI values were slightly less than the normative value of +4 reported by Redmond et al., who measured FPI on 619 healthy subjects [38]. The mean FPI scores in our study were 3.5 ± 2.9 (range -5 to 8) for the right-foot IMN and 2.9 ± 2.8 (range -1 to 7) for the left-foot IMN which based on our sample size is not significantly different to our controls (*p* = 0.21 and *p* = 0.87 respectively). Hagedorn et al. also in the Framingham population study did not find any association between foot posture and IMN [21].

Most of the literature states that the third interspace is more commonly affected with IMN for anatomical and biomechanical reasons [4, 6, 9, 25, 39]. Keh et al. [40] reported a slightly increased occurrence of neuroma in the second interspace, but an equal distribution of neuroma in both interspaces was reported by Mann et al. [5]. In this study approximately 41% occurred in the second interspace only, 34% in the third interspace only and 25% in both interspaces (Table 3). We did not find any relationship between FPI and IMN in a particular interspace. Therefore, the belief that IMN would more commonly occur in the third interspace in a more pronated foot as a result of hypermobility of the lateral column relative to the medial column cannot be supported by these findings. Furthermore, even though the second interspace was the most frequently affected site, cavus foot posture was not associated with formation of IMN in the second interspace.

It is reasonable to assume that individuals with a high BMI would have increased pressure in the forefoot during the propulsive phase of gait, which may traumatize plantar interspace nerves. However, in our study, there was no significant difference in mean BMI between IMN and control groups. The Johnston County study reported that there was no clear association between BMI and the presence of foot deformities [41]. However, in a systematic review by Butterworth et al., a significant association between foot pain as a result of non-specific foot disorders and increasing BMI was reported [42]. While obesity has been linked to an increase in plantar pressure measurements [43], which can lead to a more pronated foot and increase in foot pain [44, 45], in our case-control series no relationship between BMI and IMN formation was established.

A number of authors state that a lack of ankle dorsiflexion in gait increases pressure of the forefoot [10, 11, 34, 35, 46]. Barrett went as far as to recommend endoscopic gastrocnemius release as a treatment for IMN patients exhibiting ankle equinus [35]. DiGiovanni et al. studied the effect of isolated gastrocnemius tightness in a group of 34 patients with forefoot and midfoot pain versus a control group of 34 without any foot or ankle pain. In his study he used an equinometer to measure ankle dorsiflexion and defined equinus as less than 5°, and found that in the patient group, there was a twofold higher rate of equinus compared to the control group. The study, however, did not have any patients with IMN and only seven subjects were diagnosed with metatarsalgia of non-neurological origin. Although the measurement technique used in our study has been reported in the literature as unreliable [47, 48] the intra-rater reliability was found to be high.

One limitation of this study was that FPI is a static measurement and may not represent a subject’s dynamic function during gait given that IMN symptoms occur mostly during the propulsive phase of gait. However, some studies support the use of FPI as a valid tool in predicting dynamic function of the foot during gait [49, 50]. Secondly, gender imbalance may have affected the results, as 82% of the study subjects were female. However, there were no significant differences in mean ankle dorsiflexion measurements between male and female subjects. Presentation of IMN is more commonly seen in women, and their rate of hospital admission is three times higher that of males in Australia [1].

## Conclusion

This study examined the relationship of FPI, BMI and ankle dorsiflexion with IMN and to the author’s best knowledge is the only case-control study of this type in the literature. No relationship was found between foot type, BMI or IMN; nor was there an association between the FPI and the interspaces affected by IMN. However, there was a strong association between the presence of IMN and a restriction of ankle dorsiflexion. The authors suggest that future studies investigate the effect of the management of ankle equinus on IMN treatment.
